# Supplementary material for: Ecohydrology of urban trees under passive and active irrigation in a semiarid city
Source: PLoS One. 2019 Nov 8;14(11):e0224804. doi: 10.1371/journal.pone.0224804 (PMC6839877; doi:10.1371/journal.pone.0224804)
Supplement: Attachment — Submitted filename: Review-PONE.pdf [file pone.0224804.s001.pdf]

Article Review:

Ecohydrology of urban trees under passive and active irrigation in a semiarid city

Recommendation:

Major Revision

General comments:

In the manuscript, the authors used controlled experiment to understand how passive and active irrigation influenced urban trees in the semi-arid city. The overall research and experimental design are complete, and the data analysis and results provide useful insights to the existing literature. However, I would recommend the authors further improve all the figures quality to better support your explanation and revisit the regression analysis to make sure it explains well to the readers. A few typos and grammar errors need to be fixed as well. More detailed comments can be found in the specific comments.

Specific comments:

L48: Can you explain what types of ecosystem services are provided by the green infrastructure? This is too general. Some useful literature is attached here:

Yang, J., & Wang, Z.-H. (2017). Planning for a sustainable desert city: The potential water buffering capacity of urban green infrastructure. *Landscape and Urban Planning*, *167*, 339–347. https://doi.org/10.1016/j.landurbplan.2017.07.014

Zhao, Q., Yang, J., Wang, Z.-H., & Wentz, E. (2018). Assessing the Cooling Benefits of Tree Shade by an Outdoor Urban Physical Scale Model at Tempe, AZ. *Urban Science*, *2*(1), 4. https://doi.org/10.3390/urbansci2010004

Tzoulas, K., Korpela, K., Venn, S., Yli-Pelkonen, V., Kaźmierczak, A., Niemela, J., & James, P. (2007). Promoting ecosystem and human health in urban areas using Green Infrastructure: A literature review. *Landscape and Urban Planning*, *81*(3), 167–178. https://doi.org/10.1016/j.landurbplan.2007.02.001

L53: change “select” to “selected”

L81: similar?? Need more explanation.

L85-87:

Research goals need to be a bit clear. You only say this research applies a framework to the system, which is not very clear. Can you better describe your research objective?

L95: You would also like to explain in one sentence about how your research will contribute to the literature and real-world practise.

L112: Can you make the climate data website as a reference?

L122: What is the highlight for?

L188: Normalized greenness value is more accurate. Normalized value is too general.

L205: Where here is the data analysis section? I don't understand.

L221: medians of what?

L235-237: What is the p-value here?

L277: change R to R2

L278: the? Or they?

Table 1:

You use acronym quite frequently in your manuscript, such as PI and AI. Please make sure you explain it when it first shows up in the manuscript. Please check all your acronym again.

You might want to change the word "slope" to "coefficient"

Many of your R2 results are very low, and it almost shows no relationship. Also, can you add the p-value for your simple linear regression models?

Although you only have one parameter, you also need to explain what your dependent variable is and what is your independent variable.

I don't understand your GE part, did you run a linear regression as well here?

Figure:

Two general problems:

1. Figure dpi is too low. You need to increase the figure resolution to at least 300 dpi
2. Figure caption is too long. You can either add them into the main text or make it as a legend in the figure.

Figure 2:

Figure (a) and (b) is quite a crude map. Can you add a city boundary for Tucson? Figure (a) can be a very small inset map next to Figure (b).

You can see an example in the Figure 1 of the listed reference:

Zhao, Q., Yang, J., Wang, Z.-H., & Wentz, E. (2018). Assessing the Cooling Benefits of Tree Shade by an Outdoor Urban Physical Scale Model at Tempe, AZ. *Urban Science*, *2*(1), 4. https://doi.org/10.3390/urbansci2010004

Can you highlight the trail in the figure (c)? That will give reader a better idea.

Figure (d) and (e):

Why don't you use different name for active and passive irrigation site?

Figure 3: I saw there are some gaps in the VWC in 2017 (similar in Figure 5) and LAI in the late 2018. Why does it happen? Does the gap influence the analysis results?

Figure 6: I would recommend to separate summer and spring figures to make the x-axis clearer. And it looks like you have different intervals for your x-axis, I would recommend using the same interval for all the figures.

Figure 7: you will need a legend for this figure, especially when you have different symbols in the same figures.
