# Supplementary material for: Ecohydrology of urban trees under passive and active irrigation in a semiarid city
Source: PLoS One. 2019 Nov 8;14(11):e0224804. doi: 10.1371/journal.pone.0224804 (PMC6839877; doi:10.1371/journal.pone.0224804)
Supplement: Attachment — Submitted filename: Response to Reviewers.docx [file pone.0224804.s002.docx]

Dear Dr. Wang,

Thank you so much for your time and consideration in reviewing our submission for the PLOS One Urban Ecosystems Collection call for papers. We feel that our manuscript, “Ecohydrology of urban trees under passive and active irrigation in a semiarid city” (PONE-D-19-21151) was greatly helped by your and the reviewers’ comments.

In the pages below, we address the feedback that we received throughout the revision process. For every point raised, details are provided about how we addressed the issue and where these corrections show up in the revised manuscript. Original comments by the reviewers were pasted from the original review email, and we then provide our rebuttals in blue font color. These responses appear following the ‘Response to Reviewers’ header on the next page. Although we do not address them specifically in the next pages, additional corrections to our submission took care of any formatting issues and CC-BY permission issues for our copyrighted map images.

Thank you for the opportunity to help us share our research.

Sincerely,

Anthony M. Luketich

**Response to reviewers**

**Original comments from the academic editor and reviewers are pasted below in black ink.
Author responses and rebuttals are pasted in blue ink.**

1. Is the manuscript technically sound, and do the data support the conclusions?

The manuscript must describe a technically sound piece of scientific research with data that supports the conclusions. Experiments must have been conducted rigorously, with appropriate controls, replication, and sample sizes. The conclusions must be drawn appropriately based on the data presented.

Reviewer #1: Yes

Reviewer #2: Yes

2. Has the statistical analysis been performed appropriately and rigorously?

Reviewer #1: No

Reviewer #2: No

Both reviewers commented about the lack of statistical testing and of p-values. These have now been added throughout the manuscript including to our Table 1 of linear regression statistics.

3. Have the authors made all data underlying the findings in their manuscript fully available?

The [PLOS Data policy](http://www.plosone.org/static/policies.action#sharing) requires authors to make all data underlying the findings described in their manuscript fully available without restriction, with rare exception (please refer to the Data Availability Statement in the manuscript PDF file). The data should be provided as part of the manuscript or its supporting information, or deposited to a public repository. For example, in addition to summary statistics, the data points behind means, medians and variance measures should be available. If there are restrictions on publicly sharing data—e.g. participant privacy or use of data from a third party—those must be specified.

Reviewer #1: No

Reviewer #2: Yes

My data has now been submitted to the Dryad public repository as: Luketich, Anthony (2019), Ecohydrology of urban trees under passive and active irrigation in a semiarid city, v2, Dryad, Dataset, <https://doi.org/10.5061/dryad.m0cfxpnzf>

4. Is the manuscript presented in an intelligible fashion and written in standard English?

PLOS ONE does not copyedit accepted manuscripts, so the language in submitted articles must be clear, correct, and unambiguous. Any typographical or grammatical errors should be corrected at revision, so please note any specific errors here.

Reviewer #1: Yes

Reviewer #2: Yes

5. Review Comments to the Author

Please use the space provided to explain your answers to the questions above. You may also include additional comments for the author, including concerns about dual publication, research ethics, or publication ethics. (Please upload your review as an attachment if it exceeds 20,000 characters)

Reviewer #1: The paper is an experimental study on the impact of passive and active irrigation on urban trees. The experiment is well designed and the approach is scientifically sound. Overall the paper is well written and the results are clearly discussed. I only have minor comments:

1) For calculating the Sap velocity, Equation (2) seems to be an empirical relation. Please provide necessary references or clarifications.
I added clarification on line 179 about source material for equations 1 and 2, which includes the sensor manual and a published review of the Granier thermal dissipation method.

2) Figure 6, the R2 values are quite small in some cases, are these linear relationships statistically significant?
Statistics were ran on all regressions to find p-values. These are now listed in table 1 as well as where relevant in text and captions (i.e. fig 7)

3) As this is an experimental study, photos showing the actual in-situ instruments are highly preferred.
A methods figure (Figure 3) was added to show some installation and instruments.

4) It might be worthy to include some references on urban irrigation in semi-arid environment, such as Yang and Wang 2015 Optimizing urban irrigation schemes for the trade-off between energy and water consumption, Volo et al. 2015 An ecohydrological approach to conserving urban water through optimized landscape irrigation schedules.
Based on your comment here, and another reviewer’s comment, additional references including Yang and Wang were added. This is reflected throughout the very first intro paragraph starting on line 47

Reviewer #2: Article Review:
Ecohydrology of urban trees under passive and active irrigation in a semiarid city

Recommendation:
Major Revision

General comments:
In the manuscript, the authors used controlled experiment to understand how passive and active irrigation influenced urban trees in the semi-arid city. The overall research and experimental design are complete, and the data analysis and results provide useful insights to the existing literature. However, I would recommend the authors further improve all the figures quality to better support your explanation and revisit the regression analysis to make sure it explains well to the readers. A few typos and grammar errors need to be fixed as well. More detailed comments can be found in the specific comments.

Specific comments:
L48: Can you explain what types of ecosystem services are provided by the green infrastructure? This is too general. Some useful literature is attached here:

Yang, J., & Wang, Z.-H. (2017). Planning for a sustainable desert city: The potential water buffering capacity of urban green infrastructure. Landscape and Urban Planning, 167, 339–347. <https://doi.org/10.1016/j.landurbplan.2017.07.014>

Zhao, Q., Yang, J., Wang, Z.-H., & Wentz, E. (2018). Assessing the Cooling Benefits of Tree Shade by an Outdoor Urban Physical Scale Model at Tempe, AZ. Urban Science, 2(1), 4. <https://doi.org/10.3390/urbansci2010004>

Tzoulas, K., Korpela, K., Venn, S., Yli-Pelkonen, V., Kaźmierczak, A., Niemela, J., & James, P. (2007). Promoting ecosystem and human health in urban areas using Green Infrastructure: A literature review. Landscape and Urban Planning, 81(3), 167–178. <https://doi.org/10.1016/j.landurbplan.2007.02.001>

Based on your comment here, and another reviewer’s comment, additional references were added that talked about ecosystem services and the resulting tradeoff of water use via irrigation. The references provided were also added on line 45

L53: change “select” to “selected”
I reworded this to read more clearly on line 52: i.e. passive irrigation in “some cases” can replace active

L81: similar?? Need more explanation.
For the paragraph talking about ‘timing’ of moisture, I reworded it for clarity (line 77). Irrigation can provide moisture to plants for long periods of time irrespective of natural precipitation. Parallel to this in natural systems includes A) plants that receive more precipitation in one year (such as a wet spring) vs. another year and B) plants that have perennial groundwater access vs. plants that don’t. It gives an ecological background about moisture temporal dynamics to what can then be applied in the context of irrigation.

L85-87:
Research goals need to be a bit clear. You only say this research applies a framework to the system, which is not very clear. Can you better describe your research objective?
I rewrote this final introductory paragraph starting on line 85 to make it clearer what the goal of our observations were: to see how differences in soil moisture via differences in irrigation differentially affected the trees’ canopy and water-use metrics. I also explain our conceptual figures more clearly to show visually how we formulated our hypotheses.

L95: You would also like to explain in one sentence about how your research will contribute to the literature and real-world practise.
Done. On line 102

L112: Can you make the climate data website as a reference?
Done. On line 116

L122: What is the highlight for?
This was a mistake and is now fixed as a reference (line 130)

L188: Normalized greenness value is more accurate. Normalized value is too general.
This was reworded (line 205)

L205: Where here is the data analysis section? I don’t understand.
The data analysis section (line 223) was improved and expanded to include how we processed data, defined seasons, defined additional metrics (GE), ran statistics etc.
L221: medians of what?
The wording here (and throughout much of the results) was a bit redundant. The median comment was replaced with more concise wording (line 247).
L235-237: What is the p-value here?
p-value (<0.01) is now listed after this sentence on line 262
L277: change R to R2
Done
L278: the? Or they?
they, thanks

Table 1:
You use acronym quite frequently in your manuscript, such as PI and AI. Please make sure you explain it when it first shows up in the manuscript. Please check all your acronym again.
This was better explained in the methods section, whereby PI denotes the passive irrigation site and AI is the active irrigation site. Clearer wording is now presented at the first mention of the two different sites on line 132
You might want to change the word “slope” to “coefficient”
Because we talk about GE in terms of how the slope can denote higher or lower GE values, we left this wording as it was. However, we appreciate the clarification.
Many of your R2 results are very low, and it almost shows no relationship. Also, can you add the p-value for your simple linear regression models?
While many of our R2 results are low, some show a much stronger relationship than others and we feel it can still be a decent illustration of our comparisons between weaker vs. stronger relationships. All of the p-values were added to Table 1
Although you only have one parameter, you also need to explain what your dependent variable is and what is your independent variable.
Dependent variables noted in table caption i.e. “y as a function of x”

I don’t understand your GE part, did you run a linear regression as well here?
While the table comes first in the manuscript, GE is eventually explained in subsequent sections (line 322) but more importantly is now introduced in our data analysis section.

Figure:
Two general problems:
1. Figure dpi is too low. You need to increase the figure resolution to at least 300 dpi
Figure was changed slightly to be more concise. DPI was increased to 300.

2. Figure caption is too long. You can either add them into the main text or make it as a legend in the figure.
Figure caption was condensed for brevity. To make up for this, as well as make framework clearer for manuscript as a whole, additional lines were added to the intro starting on line 91.

Figure 2:
Figure (a) and (b) is quite a crude map. Can you add a city boundary for Tucson? Figure (a) can be a very small inset map next to Figure (b).
You can see an example in the Figure 1 of the listed reference:
Zhao, Q., Yang, J., Wang, Z.-H., & Wentz, E. (2018). Assessing the Cooling Benefits of Tree Shade by an Outdoor Urban Physical Scale Model at Tempe, AZ. Urban Science, 2(1), 4. <https://doi.org/10.3390/urbansci2010004>

Can you highlight the trail in the figure (c)? That will give reader a better idea.
Figure (d) and (e):
Why don't you use different name for active and passive irrigation site?
An improved version of the site map is now included, including a highlighted trail for (c). As far as the names, PI site vs. AI site was chosen for simplicity. Improvements were made in denoting them here, including what the acronyms stand for and individual tree names now match the sites.

Figure 3: I saw there are some gaps in the VWC in 2017 (similar in Figure 5) and LAI in the late 2018. Why does it happen? Does the gap influence the analysis results?
I addressed this in line 230, gaps should not affect analysis. The gaps were from faulty sensors or lack of access to the sites (LAI photos).

Figure 6: I would recommend to separate summer and spring figures to make the x-axis clearer. And it looks like you have different intervals for your x-axis, I would recommend using the same interval for all the figures.
(now Fig 7): Separating spring and summer seasons was done. As well as more clear and consistent axes intervals etc. Grayscale colors were changed for consistency throughout paper (as PI/gray and AI/black).

Figure 7: you will need a legend for this figure, especially when you have different symbols in the same figures.
(now Fig 8): A legend was added. Also, the grayscale colors were changed for consistency similar to the previous figure.
